# Peripherally-induced Movement Disorders: An Update

**DOI:** 10.5334/tohm.758

**Published:** 2023-03-28

**Authors:** Abhishek Lenka, Joseph Jankovic

**Affiliations:** 1 Parkinson’s Disease Center and Movement Disorders Clinic, Department of Neurology, Baylor College of Medicine, Houston, Texas, USA

**Keywords:** Movement disorders, peripheral, trauma, involuntary, dystonia, tremor

## Abstract

**Background:**

Peripherally-induced movement disorders (PIMD) should be considered when involuntary or abnormal movements emerge shortly after an injury to a body part. A close topographic and temporal association between peripheral injury and onset of the movement disorders is crucial to diagnosing PIMD. PIMD is under-recognized and often misdiagnosed as functional movement disorder, although both may co-exist. Given the considerable diagnostic, therapeutic, and psychosocial-legal challenges associated with PIMD, it is crucial to update the clinical and scientific information about this important movement disorder.

**Methods:**

A comprehensive PubMed search through a broad range of keywords and combinations was performed in February 2023 to identify relevant articles for this narrative review.

**Results:**

The spectrum of the phenomenology of PIMD is broad and it encompasses both hyperkinetic and hypokinetic movements. Hemifacial spasm is probably the most common PIMD. Others include dystonia, tremor, parkinsonism, myoclonus, painful leg moving toe syndrome, tics, polyminimyoclonus, and amputation stump dyskinesia. We also highlight conditions such as neuropathic tremor, pseudoathetosis, and *MYBPC1*-associated myogenic tremor as examples of PIMD.

**Discussion:**

There is considerable heterogeneity among PIMD in terms of severity and nature of injury, natural course, association with pain, and response to treatment. As some patients may have co-existing functional movement disorder, neurologists should be able to differentiate the two disorders. While the exact pathophysiology remains elusive, aberrant central sensitization after peripheral stimuli and maladaptive plasticity in the sensorimotor cortex, on a background of genetic (two-hit hypothesis) or other predisposition, seem to play a role in the pathogenesis of PIMD.

## Introduction

Peripherally-induced movement disorder (PIMD) is a group of conditions manifested by involuntary movements or other motor abnormalities that are induced by or emerge in the context of injury to the peripheral nervous system [1,2,3,4]. The first description of PIMD dates back to 1899 when Gowers reported a case of writer’s cramp that presumably emerged after wrist sprain [5]. Since then, several case reports and series have expanded the phenomenology of PIMD. The spectrum of the phenomenology of PIMD is broad, encompassing both hyperkinetic and hypokinetic movement disorders [2,4]. There is no confirmatory test to ascertain whether a movement disorder is genuinely induced by peripheral injury, and neurophysiological studies are rarely helpful. Therefore, the diagnosis usually rests on a strong temporal association between the injury and the onset of the movement disorders in body parts that are neuroanatomically contiguous with the location of the initial injury. Examples of peripheral injuries that may lead to PIMD include direct trauma, crush injuries, and laceration. While most of the literature is on accidental peripheral injury, it must be emphasized that any alteration of normal anatomy and physiology may result in PIMD. Therefore, surgical procedures and limb immobilization have been included under the umbrella term “peripheral trauma” as they have been reported to be linked to PIMD in some patients. In addition, though rare, there are reports of movement disorders arising de novo after electrocution or lightning, which may be considered as one of the “peripheral” triggers [6,7]. The other categories of movement disorders that may be considered as “peripherally-induced” are some of the task-specific movement disorders that may occur after repetitive activities, sometimes referred to as overuse syndrome [8,9]. Examples include musician’s dystonia, sports-related dystonia, and other movement disorders in performing artists, which often emerge in the context of years of highly precise skillful motor tasks or a history of a recent increase in practice [10,11,12]. One key sensory association with PIMD is pain. Several reports have provided evidence of a clinical and pathogenic link between PIMD and complex regional pain syndrome (CRPS) [13,14,15].

Despite numerous clinical reports, the precise pathophysiological correlates of PIMD have not been completely understood. Although hindered by significant issues such as recall bias and unavailability of appropriate control groups, basic, animal, and clinical research continue to advance the understanding of the putative pathogenesis of PIMD. As PIMD often results in a substantial worsening of the quality of life, it is crucial to revisit this topic in order to obtain better insights into its phenomenology, pathogenesis, and management.

In this invited review article, we comprehensively discuss the phenomenology, associated features, diagnostic conundrums, and putative pathophysiological substrates of PIMD and provide directions for future research and treatment strategies in PIMD.

## Method of Literature Review

The authors performed a literature search in PubMed in February 2023. Articles published in the English language were screened by combing the terms “Peripherally induced,” “peripherally triggered,” and “peripheral nervous system” with several keywords representing various phenomenologies such as “movement disorders,” “hyperkinetic,” “hypokinetic,” “parkinsonism,” “spasm,” “dystonia,” “tremor,” “myoclonus,” “myoclonic,” “chorea,” “choreiform,” “choreic,” “athetosis,” “ballismus,” “ataxia,” and “myorhythmia.” Articles were selected for a detailed review on the basis of relevance and originality with regard to the theme of the current article. References of the selected articles were searched to obtain additional articles relevant to the theme of the current topic.

## Diagnostic Criteria and Phenomenology of Peripherally-Induced Movement Disorders

Cardoso and Jankovic proposed the diagnostic criteria for PIMD, which has three core components: (a) *severity of the trauma* (should be severe enough to cause local symptoms for at least two weeks or requires medical evaluation within two weeks after trauma, (b) *location of the trauma* (body part with the involuntary movements should be anatomically related to the site of trauma, and (c) *onset of the movement disorder* (should be within days or months, up to one year after the injury) [2,16]. These criteria have been a matter of scientific debate for several reasons, especially regarding the subjectiveness of the “severity” of trauma and the latency of up to one year between the initial trauma and the onset of the movement disorders [17,18]. One of the challenging issues related to PIMD is how to differentiate this disorder from those of functional origin. As PIMD and functional movement disorders may coexist in some patients, up to 15% in one study [4], we offer some tips that may be helpful in distinguishing these two entities (Table 1). Nonetheless, the criteria mentioned above continue to provide a framework for the diagnostic workup for PIMD. They, however, do not apply to all PIMD, such as hemifacial spasm (HFS), which has been attributed to irritation or compression of the facial nerve by an aberrant blood vessel or other structures in the middle and posterior fossa, as well as many other causes [19].

**Table 1:** Summary of the key features that distinguish peripherally induced movement disorders from functional movement disorders.


FEATURES	PERIPHERALLY-INDUCED MOVEMENT DISORDERS	FUNCTIONAL MOVEMENTDISORDERS

**History of local peripheral trauma/surgery/immobilization**	**+++**	**+/–**

**Presence of psycho-social stress**	**+/–**	**++**

**Abrupt onset of symptoms**	**+/–**	**+++**

**Waxing and waning of symptoms**	**–**	**++**

**Distractibility**	**–**	**++**

**Entrainability**	**–**	**++**

**Suggestibility**	**–**	**++**

**Association with CRPS**	**++**	**–**

**Association with pain**	**++**	**–**

**Spread of movements to other body parts**	**+**	**++**

**Litigation/worker’s compensation**	**++**	**++**


**CRPS:** Complex regional pain syndrome.

**Figure 1:**
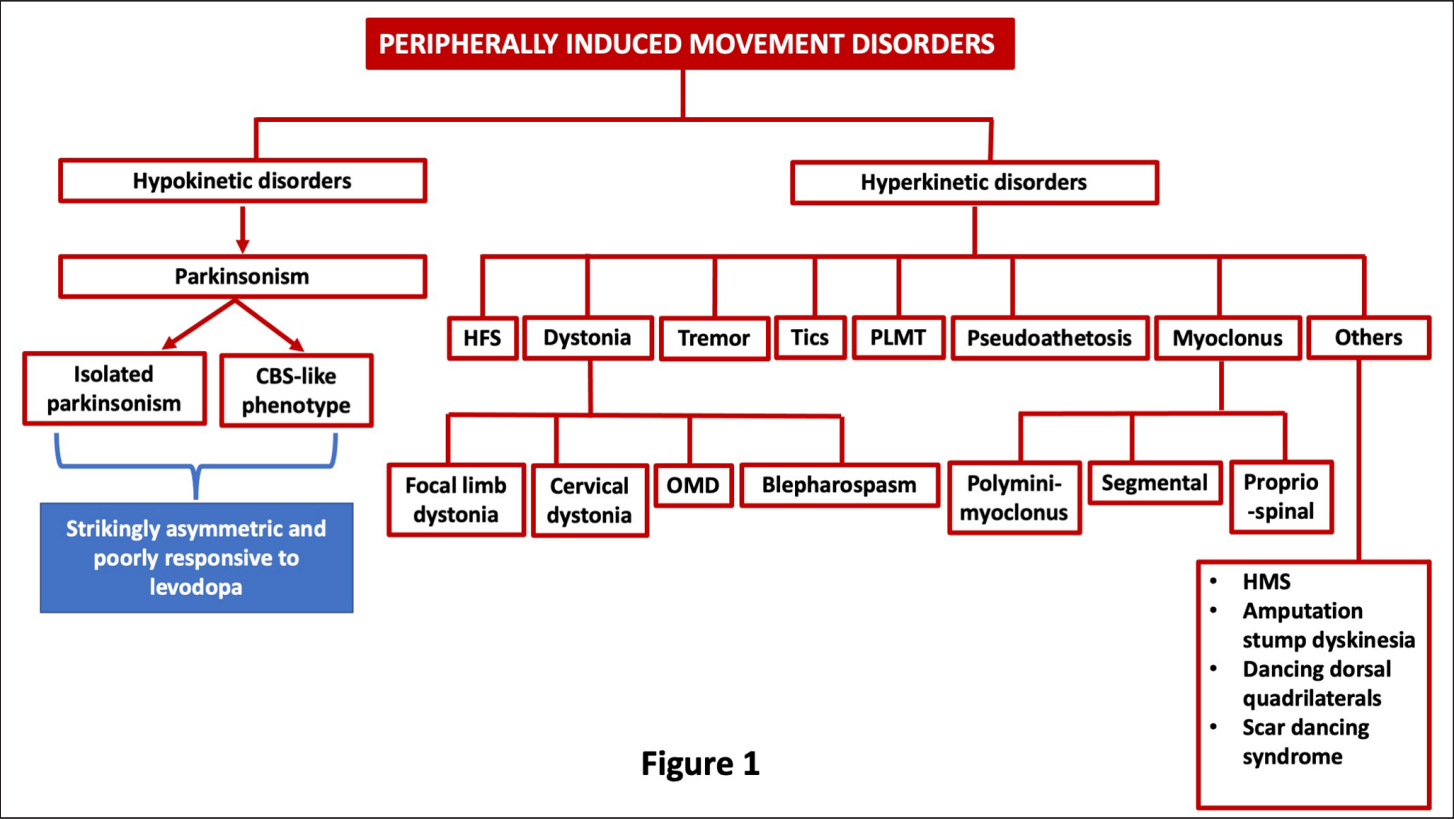
Phenomenology of peripherally-induced movement disorders. CBS: corticobasal syndrome, HFS: Hemifacial spasm, HMS: Hemimasticatory spasm, OMD: oromandibular dystonia, PLMT: Painful leg moving toe syndrome.

Over the years, numerous case reports and series have widened the spectrum of the phenomenology of PIMD. With the possible exception of HFS, dystonia is the most common phenomenology in patients diagnosed with PIMD (Figure 1). Other phenomenologies include tremor, parkinsonism, myoclonus, tics, hemimasticatory spasms (HMS), painful leg moving toes (PLMT), and synkinesis [2,4]. Sometimes, additional features such as apraxia on the side of the trauma or immobilization could emerge, which, in addition to parkinsonism, may lead to a diagnosis of corticobasal syndrome (CBS) [20]. Below, we have summarized the key aspects of some of the commonly observed PIMD.

### Hemifacial and Hemimasticatory spasm

HFS is probably the most common PIMD. Since the key mechanism of HFS is irritation of the facial nerve, a part of the peripheral nervous system, this movement disorder is regarded as a PIMD. It is characterized by involuntary, intermittent, irregular, tonic, or clonic contractions of muscles innervated by the ipsilateral facial nerve [19]. HFS is usually unilateral; there are rare reports of bilateral HFS [21,22]. Although the majority of HFS are idiopathic, presumably due to irritation or compression of the facial nerve by an aberrant blood vessel in the middle or posterior fossa or other vascular condition (e.g., arteritis), there are other causes of facial nerve compression, including tumors [19,23]. HFS may also be present in children; however, it is rare and is usually associated with vascular or tumor etiology [24,25]. Especially common in people of Asian origin, HFS can also occur in families, although disease-specific gene mutation has not yet been identified [26,27]. Neurologists should be familiar with other conditions that may mimic HFS. These include functional facial spasms, facial tics, facial dystonia, and facial myoclonus [19]. In a study from our center, lack of “other Babinski sign,” tonic muscle contractions, and downward deviation of angle of mouth had a high sensitivity for distinguishing organic HFS from functional (psychogenic) HFS [28]. The presence of additional focal neurologic or systemic signs should prompt a detailed workup to rule out secondary causes.

HMS may resemble HFS, but in contrast to facial nerve irritation or compression in HFS, the motor trigeminal nerve is affected in HMS [29]. Much less common than HFS, HMS is characterized by unilateral, involuntary, intermittent contractions of the jaw-closing muscles, resulting in brief twitches and/or spasms without actual jaw deviation [30,31]. Patients with HMS may have additional features such as facial hemiatrophy, connective tissue disorders such as scleroderma, and skin changes such as morphea [32,33,34].

HFS and HMS usually markedly improve with botulinum toxin injection, especially when performed by experienced clinicians [35,36,37]. In addition, if there is a definite neurovascular compression, HFS and HMS may improve after surgical vascular decompression of facial or trigeminal nerve, respectively [38,39].

### Dystonia

Dystonia is one of the most commonly reported phenomenologies in patients with PIMD. In a systematic review, Van Rooijen and colleagues reported that the prevalence of dystonia among patients with PIMD was 72% [4]. Since fixed dystonia, a typical presentation of functional (psychogenic) dystonia, is more common than mobile dystonia after peripheral injury, some patients with peripherally-induced dystonia have been diagnosed with functional movement disorder [40,41,42]. Some key features of peripherally-induced dystonia include marked restriction of the range of movements, sometimes associated with contractures, lack of alleviating maneuvers (sensory tricks), frequent association with CRPS, and lack of a good response to botulinum toxin [2]. Pain is a common feature in patients with post-traumatic dystonia. Dystonia occurs in 20-30% of cases of CRPS, and in such cases, dystonia is usually fixed in nature [4,13]. There is wide variability in the duration between the onset of CRPS and the emergence of dystonia in the first affected extremity (less than a week to up to a year). Compared to those without CRPS, patients with dystonia in the context of CRPS are usually younger and have a greater risk of the spread of dystonia in the future [4,13]. One of the major risk factors for peripherally-induced limb dystonia is prolonged immobilization (termed “immobilization dystonia”), such as casting after a bone fracture. Okun and colleagues drew attention to this phenomenon in a series of four patients who developed dystonia after weeks of cast immobilization [43]. Only two of the four patients had associated pain, and none had CRPS. Thus while pain and CRPS are often associated with peripherally-induced dystonia, these elements are not always present [43].

Cervical dystonia (CD) has been commonly reported in the context of neck trauma and thus may be considered a form of peripherally-induced dystonia. In a series of 114 patients with CD, Sami et al. reported pre-existing head/neck injury (within one year of the onset of CD) in 12% of the patients. In that series, there were no striking clinical differences between patients with CD with and without pre-existing trauma [44]. Clinical features, however, may vary depending on the interval between trauma and the onset of dystonia. For example, patients with acute onset CD, compared to those with delayed-onset CD, have severely restricted cervical range of motion, more pain, higher incidence of shoulder elevation and laterocollis, lack of phasic involuntary movements, and poor response to botulinum toxin and physical therapy [45,46,47,48]. Conversely, patients with delayed-onset CD are often clinically indistinguishable from those with idiopathic CD [49].

Other peripherally-induced focal dystonia include blepharospasm and oromandibular dystonia (OMD). A review of 264 patients with blepharospasm by Grandas and colleagues revealed the presence of pre-existing ocular lesions in 12.1% [50]. In addition, a strong association of ocular symptoms such as dry eyes with the occurrence of blepharospasm supports the concept of peripherally-induced blepharospasm [51]. While there are several publications on secondary blepharospasm in the context of central nervous system (CNS) lesions, peripherally-induced blepharospasm is relatively rare [52,53].

OMD has been reported in individuals after face, mouth, or jaw injury or after dental surgical procedures [54,55,56]. The clinical phenomenology of post-traumatic OMD is often indistinguishable from primary OMD, which may pose diagnostic challenges [55]. The term “edentulous dyskinesia” describes new onset involuntary movements associated with dystonia after tooth extraction [57]. In a series of 14 patients with edentulous dyskinesia, dystonia was limited to the oral region and spared the tongue [58]. Compared to other forms of post-traumatic OMD, functional disability is usually minimal in patients with edentulous dyskinesia [57,58]. It is essential to be familiar with edentulous dyskinesia as the symptoms may potentially improve with restoration procedures or fitting dentures [59]. The phenomenology of edentulous dyskinesia often overlaps with that of tardive dyskinesia [60]. It is, therefore, critical to explore the history of drug exposure, as dopamine receptor-blocking drugs (e.g., antipsychotics and antiemetics) are typically associated with tardive dyskinesia in the form of orofacial stereotypy or OMD [60]. Many other disorders should be considered in the differential diagnosis of OMD, including tics, bruxism, and others [54,61].

As mentioned above, task-specific dystonia may also be considered a category of PIMD, particularly if occurring after prolonged repetitive motor activity (“overuse syndrome”) [8,9]. Musicians and their physicians often attribute their task-specific dystonia to intense practice before an important performance, but the link with the overuse syndrome is often difficult to establish [10,11]. While sports-related dystonia often follows some limb injury, the peripheral trigger may not always be obvious. Common forms of sports-related dystonia include golfer’s dystonia (often referred to as “yips”) and “runner’s dystonia.” However, many other sports activities can be associated with task-specific dystonia, including gymnastics, ice skating, tennis, table tennis, pistol shooting, petanque, billiards, and baseball [62]. While botulinum toxin injection by an expert often alleviates task-specific dystonia, many professional athletes and musicians have their careers threatened because of persistent movement disorder.

There is a huge unmet need for long-term outcome data on patients with peripherally-induced dystonia. In general, complete or meaningful recovery is highly unusual in patients with peripherally-induced dystonia, especially those with fixed dystonia and contractures. Some patients with post-traumatic CD, OMD, blepharospasm, or task-specific dystonias may respond well to local botulinum toxin injections [35]. However, co-existing functional (psychogenic) components or overlay may complicate the clinical course [18,40,63]. When co-existing functional component is suspected, patients should be referred for treatment (not diagnosis) to experienced psychologists and psychiatrists.

A few centers have used deep brain stimulation (DBS) or ablative lesions in the thalamus in treating post-traumatic and task-specific dystonia [64,65]. Results have been disappointing in the former and encouraging in the latter [66,67,68,69]. However, reports on these surgical interventions in PIMD are limited in number and methods of assessment.

Table 2 highlights the key differences between peripherally-induced dystonia and idiopathic dystonia.

**Table 2:** Summary of the key features that distinguish peripherally induced dystonia from idiopathic dystonia.


FEATURES	PERIPHERALLY INDUCED DYSTONIA	IDIOPATHIC DYSTONIA

**Onset of symptoms**	Acute/subacute	Insidious

**Pain**	**+++**	**+**

**CRPS**	**++**	**-**

**Persistence during sleep**	**+**	**-**

**Fixed dystonia**	**++**	**-**

**Type of dystonia**	Tonic	Phasic + Tonic

**Response to alleviating maneuvers (sensory tricks)**	**-**	**++**

**Response to botulinum toxin**	**-**	**++**

**Response to DBS**	**-**	**+** (depends on the type and etiology of dystonia)


**CRPS:** Complex regional pain syndrome, **DBS:** Deep brain stimulation.

### Tremor

Post-traumatic tremor is the most common movement disorder caused by brain injury [70,71], but peripherally-induced tremor is relatively uncommon. In one of the earliest reports of peripherally-induced tremors, five of 23 patients with PIMD had tremor; in one, tremor followed initial dystonia [72]. All had a unilateral hand or foot tremor except one patient with an electric shock injury who developed a bilateral hand tremor. In another series of 28 patients with dystonia and tremor after peripheral trauma, 17 had only tremor (mostly postural), and 11 had co-existing parkinsonism [16]. A family history of essential tremor was considered one of the common predisposing factors, although other genetic or environmental factors were proposed in this and other series of PIMD. There are reports of emergence of tremor after iatrogenic peripheral nerve injury (e.g., after thoracic surgery) [73] and traumatic neck injury [74]. The latter case is interesting because it provides insight into the neurophysiologic basis of peripherally induced tremor. The authors explored the effect of ischemic nerve block and torque loading on postural upper extremity tremor in a patient with a traumatic neck injury [74]. The outcome was a decrease in tremor frequency after both interventions, probably because tremor was secondary to abnormal peripheral mechanical reflex mechanisms [75].

Neuropathic tremor may be considered a form of PIMD. This form of tremor is present in some patients with severe peripheral neuropathy without other movement disorders [12]. Specific forms of peripheral neuropathy have a greater predilection for tremors than others. Patients with IgM paraproteinemic neuropathies, chronic inflammatory demyelinating polyradiculoneuropathy (CIDP), multifocal motor neuropathy with conduction block, and Charcot-Marie-Tooth (CMT) disease are especially prone to experience tremor [76,77,78]. Although the mechanisms are not well understood and probably vary from case to case, the prevalence of tremor in inflammatory neuropathies may range from 40-70% [76,78]. In a recent study of 24 patients with CIDP, 66% had an ET-like postural tremor [79]. Contrary to the presumption that tremor in neuropathy is primarily peripherally generated, the authors provide evidence for a co-existing role of a central component by demonstrating that tremor in CIDP was unaffected by weight loading [79]. Specific subtypes of CIDP, such as those associated with neurofascin155 (nfasc155) IgG4 antibodies, are particularly likely to be associated with disabling low-frequency, high-amplitude action tremor of the upper extremities [80,81].

A newly described genetic disorder named “myogenic tremor,” first reported in 2019, may be considered a form of peripherally-induced tremor [82,83]. This genetic disorder, transmitted by an autosomal dominant pattern, results from a mutation in the *MYBPC1* gene (codes for myosin binding protein). *MYBPC1* mutation affects the binding of MBPC1 to myosin, resulting in dysregulated cross-bridge cycling during sarcomeric contraction. In addition to proximal myopathy and skeletal deformities (scoliosis, thoracic deformities), all patients in the published study had postural hand tremor [83]. Patients with this condition have tremor from the neonatal period that persists until adulthood. One patient described in the first original publication on myogenic tremor had tongue tremor since neonatal life. The investigators speculate that the myogenic tremor has a sarcomeric origin which gets support from the fact that *MYBPC1* is expressed only in the skeletal muscles, and the mutation is primarily linked to dysregulated cross-bridge cycling during sarcomeric contraction [83]. The jerky component of the tremor suggests a possible link to polyminimyoclonus (discussed below). Recent neurophysiologic studies suggest that while the tremor may originate within muscles, it may engage muscle spindles to activate central mechanisms and tremor oscillators [84].

### Parkinsonism

Whether peripheral trauma precipitates parkinsonism or worsens pre-existing parkinsonism has remained controversial since the first description of Parkinson’s disease (PD) by James Parkinson [85]. Repeated head trauma and even single traumatic brain injury have been proposed as risk factors for parkinsonism [86,87,88,89], but the data on peripheral trauma triggering parkinsonism is relatively sparse. Earlier reports from our center described well-documented cases of parkinsonism developing a few days or weeks after peripheral trauma [16]. The lack of improvement with dopaminergic drugs suggests the possibility of a post-synaptic component. Many patients with PD report injury, such as motor vehicle accidents, prior to the onset of their symptoms. Such history is often dismissed because of possible recall bias and the presumption that the patients were simply unaware of any parkinsonian manifestations before the trauma. There are, however, many reports of musculoskeletal problems, especially “frozen shoulder,” before the onset of classic parkinsonian symptoms that are often attributed to local injury. While it can be argued that those are early symptoms of PD (such as decreased arm swing leading to a “frozen shoulder”), shoulder pain is now well recognized early symptom of PD [90,91,92,93,94]. Although the relationship between shoulder problems and PD is not well understood, we and others have encountered many well-documented cases of parkinsonism with onset within days or weeks following shoulder injury [16].

In one intriguing study from the Mayo Clinic, 10 out of 44 (23%) patients diagnosed with CBS had a history of limb immobilization or trauma in the most affected limb [20]. In six patients, immobilization/trauma preceded the onset of CBS symptoms, whereas four patients had pre-existing limb dysfunction that significantly worsened after immobilization/trauma. While more extensive studies are required to understand this association better, this study highlights the potential association between peripheral trauma and CBS [20]. This association is particularly intriguing because CBS is the most asymmetric of all neurodegenerative parkinsonian disorders [95]. In our clinical practice (unpublished data), we have encountered several patients who developed CBS a few days or weeks after peripheral trauma or surgery (with apraxia or dystonia on the ipsilateral side).

### Myoclonus and polyminimyoclonus

In the systematic review by van Rooijen et al. that reviewed 713 patients (from 133 publications), 95 patients (13%) had myoclonus, in isolation or combination with other movement disorders, after peripheral trauma [4]. There are reports of different types of myoclonus (segmental, propriospinal) after accidental and iatrogenic injuries [96,97,98]. Myoclonus may coexist with dystonia and tremor in some patients with CRPS [13]. Several case reports have described myoclonus emerging after peripheral nerve injury [96,99,100,101,102,103,104]. One of the most vital pieces of evidence in favor of the concept of “peripherally-induced myoclonus” is that in many patients with peripherally-induced myoclonus, nerve block with local anesthetics ameliorates the myoclonus [102,103]. It is presumed that peripheral nerve or root injury, in some individuals, triggers ephaptic transmission and ectopic excitation that could be associated with the emergence of myoclonus. This is especially relevant in cases of spinal segmental myoclonus, manifested by rhythmic, jerk-like contractions of muscles (or other dyskinesias, such as rotatory movement of the scapula or “belly dancer” dyskinesia) innervated by cervical, thoracic or lumbar roots. Such segmental movements have been described after peripheral or spinal injury, procedure, or surgery [105,106,107]. Treatment of spinal myoclonus with tetrabenazine was first described by Hoehn and Cherington [108]. We have also found the use of vesicular monoamine transporter 2 inhibitors, in addition to clonazepam, helpful in treating spinal myoclonus. Patients with bothersome peripherally-induced myoclonus may benefit from local botulinum toxin injections, which result in chemodenervation of the intrafusal muscle fibers and, in turn, reduces the spindle afferent input [96,100].

Polyminimyoclonus (also called minipolymyoclonus) is a type of involuntary hyperkinetic movement that is often described in patients with anterior horn cell diseases such as amyotrophic lateral sclerosis and brachial monomelic amyotrophy (Hirayama disease) [109]. It is characterized by intermittent, slow, small-amplitude movements of hands, especially of the digits. In a series of 77 patients with various motor neuron diseases, more than two-thirds of patients had involuntary movements of their hands [110]. Polyminimyoclonus was documented at rest and during action in 39.2% and 32.8%, respectively. Other involuntary movements were thumb tremor and pseudo-dystonic posturing of the thumb [110]. Most patients with Hirayama disease, who are typically adolescent males with initially progressive hand weakness and atrophy, also have polyminimyoclonus [111,112]. Although the precise neuroanatomical correlates are poorly understood, it is speculated that polyminimyoclonus is probably a result of frequent fasciculations, which give the appearance of myoclonic movements [111]. That is why some have proposed the term “polyminifasciculations” [109,113].

### Miscellaneous peripherally-induced movement disorders

A plethora of PIMD, other than those mentioned above, have been reported in the past, and we briefly describe those below.

Motor tics have been reported after peripheral injury [114,115,116]. These tics usually occur in adults but are phenomenologically similar to the childhood-onset tics associated with Tourette syndrome, even associated with a sensory urge prior to the movement.

Although rotatory movements of the scapula and other abnormal involuntary movements of the shoulder are typical manifestations of dystonic tics in patients with Tourette syndrome, shoulder involuntary movements may be peripherally induced. One cause of shoulder (scapular) dyskinesia is neuralgic amyotrophy [117]. This condition, manifested by episodes of acute pain in the proximal arm, presumably due to acute autoimmune inflammation of the nerves in the brachial plexus, is often associated with abnormal involuntary movements of the shoulder. Dancing dorsal quadrilaterals is a recently described syndrome manifested by repetitive, rotatory movements of the scapular region, especially the trapezius and rhomboids, occurring after spine instrumentation [118]. Another movement disorder involving the shoulder includes “scapular dyskinesis,” presumably secondary to the instability of the shoulder joint or abnormalities in the pectoralis muscle following shoulder surgery [119].

Pseudoathetosis may be considered a form of PIMD. The term “athetosis,” which has a Greek origin meaning “without a fixed position,” refers to an involuntary movement characterized by slow, writhing movements (essentially slow chorea). Although typically encountered in patients with basal ganglia lesions, athetosis may also occur in the setting of profound loss of proprioception, hence the term pseudoathetosis [120]. Since phenomenologically, it is identical to athetosis of central origin, a more appropriate term should be “proprioceptive athetosis.” Typically involving the fingers or toes, in contrast to central athetosis, the proprioceptive athetosis (pseudoathetosis) usually worsens when the eyes are closed [121]. There are many etiologies of pseudoathetosis, but it is typically caused by large fiber peripheral neuropathy [121,122,123]. Proprioception abnormalities secondary to CNS lesions, such as parietal cortex stroke [124], neuromyelitis optica [125], or cervical myelopathy [126] may also cause pseudoathetosis.

PLMT is a rare PIMD where limb pain is a key feature. In a series of 76 cases, 42% were cryptogenic, whereas in other cases, the common etiologies were peripheral neuropathy, trauma, and radiculopathy [127]. PLMT may have unilateral or bilateral leg involvement, and the onset of pain almost invariably precedes the onset of involuntary movements. While there are reports of improvement in symptoms by medications such as pregabalin [128], gabapentin [129], and clonazepam [130], the overall response to treatment is not satisfactory in the majority of cases, but local botulinum toxin injections may be beneficial [127,131]. Corresponding symptom complex in the upper extremities has been reported as “painful arm moving finger” syndrome [132]. Very rarely, patients with PLMT may have involuntary movements in the ipsilateral hand [133]. A phenomenologically similar condition, but without pain, is described as “painless leg moving toe” syndrome, which has been reported in several conditions such as multiple system atrophy [134], Wilson’s disease [135], and Parkinson’s disease [136].

Some patients may develop a variety of movement disorders after surgery, even if the surgery is not directly related to the nervous system. Some of the examples include dystonic gait after hip surgery [137], hemi-abdominal myoclonus after uterine surgery [138], dyskinesia along scar tissue which was named “scar dancing syndrome” [139], and post-amputation stump dystonia and dyskinesia [140,141]. We refer the readers to a recently published review that describes post-surgical movement disorders in detail [142].

Figure 1 summarizes the phenomenology of PIMD.

## Possible Pathophysiologic Mechanisms

While there are no established neural correlates of PIMD, the classic experiments in non-human primates by Byl and colleagues [143] provide the basis for the notion that single (e.g., amputation) or repetitive (e.g., overuse) peripheral injury results in central neuronal reorganization and altered motor activity, such as tremor or dystonia, in the affected body part [144]. Most of the evidence for this theory stems from animal models of peripheral injury. For example, there are reports of reorganization of the motor and somatosensory cortex in upper extremity amputees with phantom pain [145,146,147,148]. Loeser and Ward demonstrated that experimental deafferentiation of neurons in the spinal cord of cat (by dorsal rhizotomy or hemicordotomy) results in spontaneous hyperactivity and abnormal firing of second-order neurons [149]. Previous studies have shown that peripheral deafferentiation in cats and monkeys may result in organizational changes in the sensory neurons in the nucleus gracilis and the ventral posterior nucleus of the thalamus, respectively [150,151]. Such supraspinal aberrant changes may result in permanent changes in the somatosensory cortex. Neuroplasticity resulting in somatotopic interference has also been reported in individuals following spinal cord injury [152]. Maladaptive cortical reorganizations may not be limited only to noxious peripheral processes as they may also extend to non-noxious and task-induced inputs. For example, patients with musician’s dystonia have abnormal functional connectivity within a wide range of brain structures [153,154,155].

Experimental studies on the mouse models of *DYT1 (Tor1A)-*associated dystonia provide compelling evidence of genetic predisposition for a link between peripheral injury and dystonia [156,157]. In one study, two weeks after experimental crush injury of peripheral nerves, there was dystonic posturing of the lesioned hindlimbs in both mutant (Tor1a+/-) and wild-type mice [157]. However, the phenotypic abnormalities after the peripheral injury increased by 40% in the mutant mice than in the wild-type. Moreover, a dystonia-like phenotype was accompanied by abnormalities in the homeostasis of striatal dopamine. In another study, in addition to replicating the previous study’s findings, the same group of investigators demonstrated dopamine dysregulation and aberrant oscillatory activity of a central motor network in Tor1a rats [158]. It is, therefore, possible that in some patients with PIMD, there are some pre-existing genetic risk factors (“first hit”), and certain peripheral trauma reduced the threshold of manifestation of PIMD (“second hit”). The “two-hit” hypothesis in the context of aberrant neuroplasticity and environmental factors (such as trauma) is elegantly described in a review article by Rauschenberger and colleagues [159].

Although not dystonia, deformities associated with arthrogryposis are often confused with dystonia. This disorder manifests with congenital joint contractures and has recently received attention from the scientific community after a study found that a gain-of-function mutation in the *PIEZO2* gene is associated with congenital distal arthrogryposis [160]. PIEZO2 is a mechanically-activated ion channel and is the main mechanosensor of somatosensory neurons. The *PIEZO2* mutation causing joint deformities reinforces the notion that abnormalities in proprioception may lead to musculoskeletal deformities [160]. Interestingly, administration of botulinum toxin in the critical period rescued the limbs from the detrimental effects of *PIEZO2* gain-of-function. A similar mechanism could play a central role in peripherally-induced dystonia, and future studies should investigate the role of *PIEZO2* overexpression in patients with peripherally-induced dystonia and the putative role of early botulinum toxin injection.

Literature on the potential pathogenesis of PIMD other than dystonia is scarce, probably due to the rarity of such conditions. A recently published study explored the possibility that thoracic trauma could trigger central pathological changes in mouse models of PD [161]. The findings from this study provided evidence that peripheral injury (i.e., blunt thoracic trauma) results in an increased inflammatory response and alpha synuclein oligomerization in the brains of transgenic mice overexpressing alpha synuclein and behavioral features of parkinsonism. There was a clear increase in alpha synuclein oligomers in PD mice exposed to thoracic trauma compared to sham-treated mice. The authors suggested that “peripheral injury influences protein aggregation in the brain of PD mice” and hypothesized that “here could be an immediate ascending inflammatory reaction from the periphery that eventually reaches the CNS” [161]. While this study did not explicitly demonstrate a causal relationship between peripheral trauma and de novo parkinsonism, it supports the “two-hit” hypothesis, i.e., in predisposed individuals (first hit), trauma (second hit) results in increased alpha synuclein oligomerization. Additional studies are warranted to obtain deeper insights into the association of peripheral trauma and altered alpha synuclein neurobiology.

## Conclusion and Future Directions

PIMD is an underrecognized and underdiagnosed group of neurological disorders. Indeed, it is often misdiagnosed as a functional (psychogenic) movement disorder, partly because trauma typically triggers or is associated with the onset of both disorders. Because both disorders may co-exist, neurologists should be familiar with their phenomenology and clinical features to differentiate them and provide appropriate treatments. While PIMD has a broad phenomenological spectrum encompassing both hyperkinetic and hypokinetic movements, most of the patients have dystonia which may or may not be associated with pain and CRPS. Close topographical and temporal association between trauma or surgical procedures and the onset of the movement disorders should raise the suspicion of possible PIMD.

**Figure 2:**
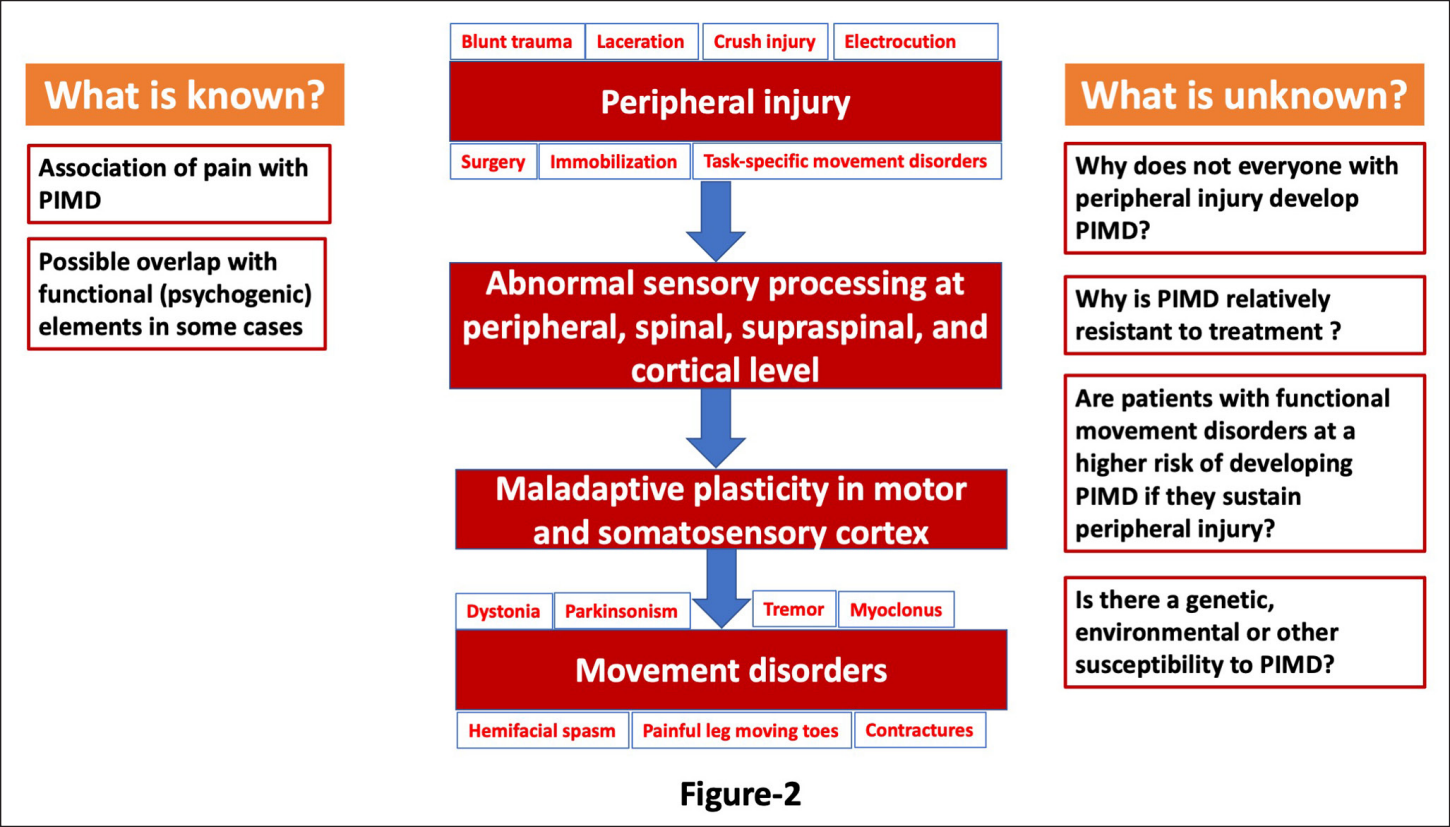
Summary of the pathogenesis of peripherally-induced movement disorders.

There are several unmet needs in the field of PIMD, including a better understanding of the pathogenesis of PIMD. To summarize current theories about PIMD, two fundamental mechanisms seem to be crucial in the pathogenesis of PIMD: 1. aberrant central reorganization in response to peripheral stimuli or injury, and 2. maladaptive plasticity in the sensorimotor cortex may alter motor output resulting in movement disorders (Figure 2). However, these two hypotheses cannot answer several questions related to PIMD. These include: 1) why does not everyone with severe injury develop PIMD?, 2) are those who develop PIMD genetically or otherwise susceptible to abnormal peripherally-induced central response?, 3) are patients with functional movement disorder at a greater risk of having PIMD and what is the relationship between the two disorders? As noted before, several factors, including recall bias and the unavailability of appropriate control groups, pose significant challenges in conducting studies on PIMD. Future studies should address these knowledge gaps, focusing on identifying predictors or biomarkers for susceptibility to PIMD and developing models that advance better understanding and treatment of PIMD.
